# The Effect of Laser Beam Processing on the Properties of WC-Co Coatings Deposited on Steel

**DOI:** 10.3390/ma14030538

**Published:** 2021-01-23

**Authors:** Norbert Radek, Janusz Konstanty, Jacek Pietraszek, Łukasz J. Orman, Marcin Szczepaniak, Damian Przestacki

**Affiliations:** 1 Faculty of Mechatronics and Mechanical Engineering, Kielce University of Technology, Al. 1000-lecia P.P. 7, 25-314 Kielce, Poland; 2 Faculty of Metals Engineering and Industrial Computer Science, AGH University of Science & Technology, 30 Mickiewicz Avenue, 30-059 Cracow, Poland; konstant@agh.edu.pl; 3 Faculty of Mechanical Engineering, Cracow University of Technology, Al. Jana Pawła II 37, 31-864 Cracow, Poland; jacek.pietraszek@mech.pk.edu.pl; 4 Faculty of Environmental, Geomatic and Energy Engineering, Kielce University of Technology, Al. 1000-lecia P.P. 7, 25-314 Kielce, Poland; orman@tu.kielce.pl; 5 Military Institute of Engineer Technology, Obornicka 136 Str., 50-961 Wrocław, Poland; szczepaniak@witi.wroc.pl; 6 Faculty of Mechanical Engineering, Institute of Mechanical Technology, Poznan University of Technology, Piotrowo 3 Str., 61-138 Poznan, Poland; damian.przestacki@put.poznan.pl

**Keywords:** WC-Co coating, ESD method, laser beam processing, pulse plasma sintering, performance characteristics

## Abstract

The main objective of the present work is to determine the effects of laser processing on properties of WC-Co electro-spark deposited (ESD) coatings on steel substrates. Tungsten carbide coatings have been applied to steel substrates using a manual electrode feeder, model EIL-8A. The laser beam processing (LBP) of electro-spark coatings was performed using an Nd:YAG fiber laser. The microstructure and properties of laser treated/melted coatings were evaluated by means of scanning electron microscopy (SEM), X-ray diffraction (XRD), surface geometric structure (SGS) and roughness measurements and adhesion, microhardness, residual stresses, corrosion resistance and application tests. The obtained experimental data were subjected to statistical analysis and multidimensional numerical and visual exploratory techniques. It has been shown conclusively that the laser-treated ESD WC-Co coatings are characterized by lower microhardness, higher resistance to corrosion, increased roughness and better adhesion to the substrate. LBP homogenizes the chemical composition, refines the microstructure and heals microcracks and pores of ESD coatings. The laser treated ESD WC-Co coatings can be used in frictional sliding nodes (e.g., on the front seal rings used in pumps) and as protective layers.

## 1. Introduction

Surface layers with a specific structure and properties are usually produced on machine elements in various processes before they are used in practice. The variety of technological possibilities leading to the products’ intended operational features is very large. Bond techniques are among the new generation of antiwear coatings. They create great technical and technological possibilities to control the parameters of surface layer manufacturing processes. Introducing them into industrial practice promises great development prospects, which in the era of globalization significantly improves the competitiveness of products.

Carbide coatings have numerous industrial applications. Characterized by high abrasion, sliding and erosion resistance, they can be used as a substitute for hard chrome plating. Cemented carbides are cermets containing between 70 and 97 wt % of refractory metal carbides (e.g., WC, TiC and TaC) and a metal binder, which is most often cobalt, sometimes nickel and occasionally an iron-based alloy. At present, cemented carbides find numerous applications as wear parts and in all kinds of machining operations outperforming the conventional high speed steel tools [1].

Cemented carbides are classified into categories depending on their chemical composition and WC grain/particle size. The latter parameter can vary widely, therefore cemented carbides are divided into four main groups [2]:

- Of coarse grains (whose diameter is 3–30 μm),
- Of standard grains (whose diameter is 1.5–3 μm),
- Of fine grains (whose diameter is 0.5–1.5 μm),
- Of ultrafine grains (whose diameter is below 0.5 μm).

The use of ceramic tool materials is limited as compared with cemented carbides, although it tends to grow. It has been estimated that around 5% of cutting tool inserts are made of ceramics based on:

- Single-phase alumina (Al_2_O_3_),
- Silicon nitride (Si_3_N_4_),
- Multiphase (Al_2_O_3_ and Si_3_N_4_) with minor additions of carbides, other nitrides and oxides.

Currently, WC-based coatings can be produced with various technologies, such as: plasma spraying [3,4], HVOF spraying [5,6], electro-spark deposited (ESD) processing [7,8], laser cladding [9,10], laser alloying [11,12], physical vapor deposition [13], chemical vapor deposition [14] and liquid state sintering in vacuum [15], which mainly fulfill protective and antiwear functions.

Among the beam techniques, we observe a dynamic development of laser processing [16,17,18] and the ESD method has a well-established position among surface treatment techniques [19,20,21].

Electro-spark deposition can be successfully used for the regeneration of elements of aircraft engines or hydraulics in special vehicles [22,23]. The production of ESD protective coatings has also found wide application in the automotive industry [24]. Many works have been devoted to assess the effect of electro-spark deposition parameters on the properties of coatings [25,26,27,28,29,30].

An interesting option is the application of antiwear coatings on steels by means of ESD treatment with electrodes obtained by pulse plasma sintering (PPS) from submicron-sized cermet powders. Super-hard coatings can also be successfully deposited on cutting edges of indexable inserts, used for turning and milling and on many other machine components, which operate under heavy-duty conditions, including extreme abrasive wear and/or impact loads.

In the as-deposited condition the ESD coatings usually have flows that can be easily eliminated by laser beam processing, wherein a laser beam is used for pore/crack sealing and surface polishing and for homogenizing the chemical composition of the coating [31,32]. Additionally, the laser-treatment aids the coating’s adhesion to the substrate, wear and seizure properties, resistance to corrosion and fatigue strength due to formation of compressive stress in the subsurface layer [33,34].

In this article the laser beam processing was carried out in order to improve the application properties of ESD WC-Co coatings. The main objective of application of a laser beam was to reduce structural defects in coatings, to develop favorable compression stresses within the subsurface layer and to improve adhesion to the substrate material and anticorrosion properties. It is assumed that the use of tungsten carbide electrodes (manufactured with the pulse plasma sintering method) will broaden the application areas of WC-Co coatings after laser processing, e.g., in drilling tools used in the mining industry or press elements in the fabrication of structural ceramics, sealing technologies, tools for plastic treatment and etc. Further research is needed to model the impact of laser treatment on the distribution of temperature fields in the heated WC-Co coating. It will be crucial for developing innovative machining technologies.

## 2. Materials and Methods

### 2.1. Coatings Production

**Figure 1:**
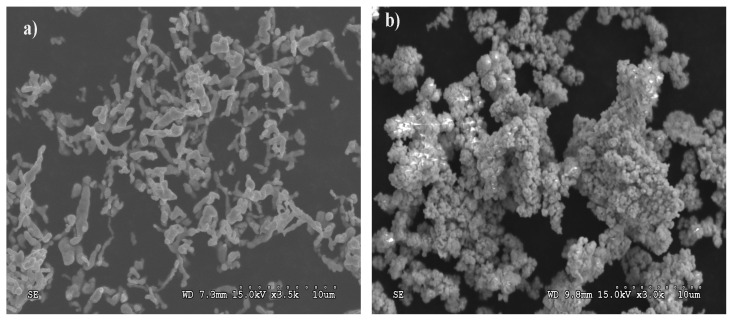
Particle morphology of submicron-grained powders: (**a**) cobalt and (**b**) tungsten carbide.

WC-Co coatings were manufactured by the ESD method. Cylindrical electrodes, 5 mm in diameter and 10 mm high, were used to deposit coatings on C45 carbon steel, N11 tool steel, HS6-5-2 and HSS-E high speed steels. The electrodes contained 95 wt % WC (OMG, Cleveland, OH, USA; FSSS = 0.2 μm) and 5 wt % Co (Umicore, Olen, Belgium; FSSS = 0.4 μm). Figure 1a,b show particle morphologies of fine cobalt and tungsten carbide powders detailed SEM revealed submicron sized grain structure of both powders.

**Figure 2:**
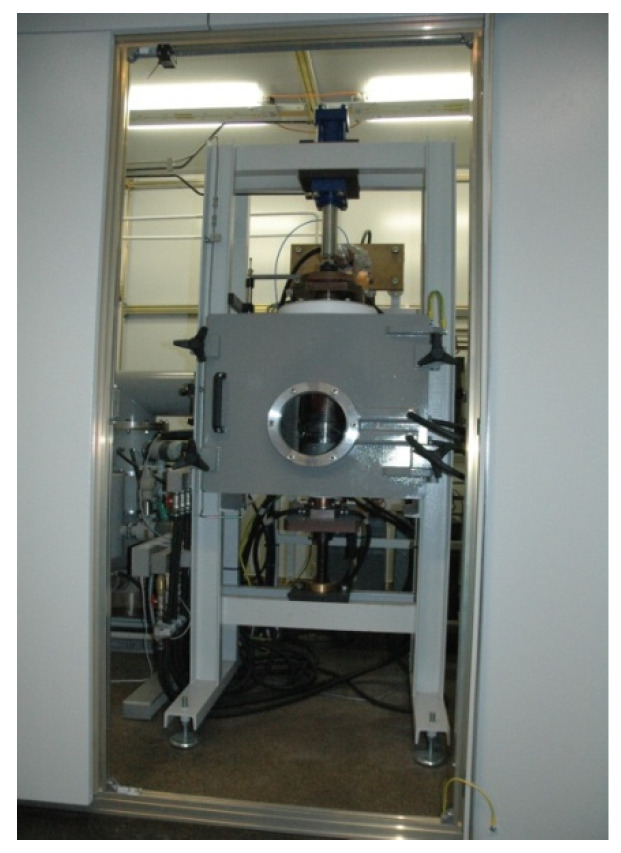
Pulse-plasma sintering (PPS) sintering facility.

**Figure 3:**
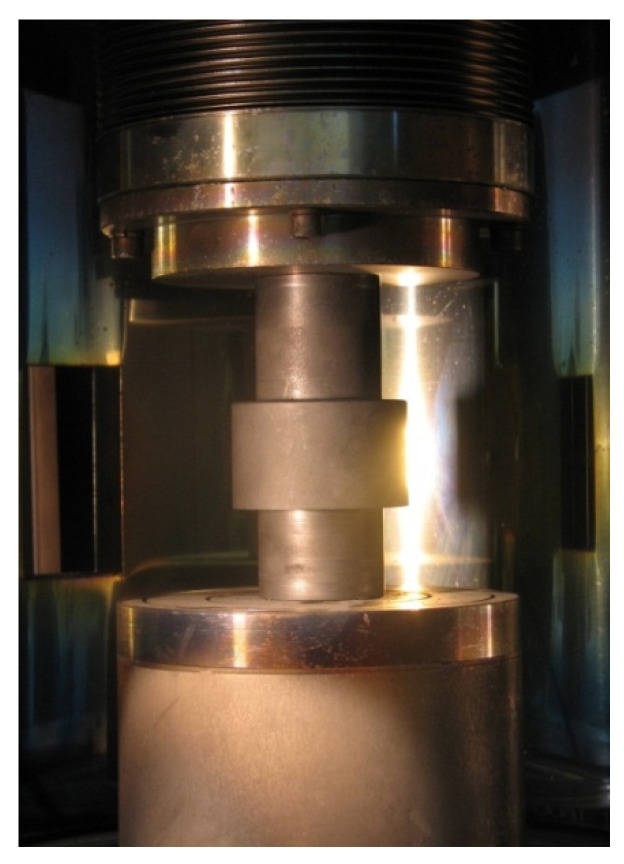
Fabrication of an electrode by PPS.

The powders were mixed together in the right proportions and consolidated by means of a pressure-assisted, pulse-plasma sintering (PPS) method at the Faculty of Material Engineering, Warsaw University of Technology (Warsaw, Poland). The powder mix was held for 5 min at 1100 °C and 50 MPa. PPS uses high-current pulses generated through continual discharging a capacitor battery of 300 μF, thereby inducing several tens of kA current, which flows through the consolidated powder within each millisecond pulse. Figure 2 and Figure 3 present the PPS sintering facility and a general view of the electrode fabrication setup, respectively.

The coatings deposition was performed in argon by means of the EIL-8A pulse generator with manual electrode displacement. Following the manufacturer’s guidelines and using prior experience, the voltage, capacitor volume, current intensity and deposition time were set to 230 V, 300 μF, 2.1 A and 2 min/cm^2^, respectively.

The coatings were subsequently subjected to laser beam processing (LBP) at the Centre for Laser Technology of Metals, University of Technology, Kielce. The Basel Lasertechnik (Düren, Germany) BLS 720 Nd:YAG laser generated a beam having 1 mm spot diameter and 25 W power was operated in the pulse mode. The laser treatment conditions were:

- Pulse duration time: 0.5 ms;
- Frequency: 45 Hz;
- Stroke of laser beam: 0.35 mm;
- Speed of movement: 230 mm/min.

### 2.2. Characterization of Coatings

The morphology of WC-Co coatings was analyzed before and after laser treatment by means of the Quanta 3D FEG (SEM/FIB) scanning electron microscope, equipped with an integrated EDS/WDS/EBSD system (energy dispersion spectrometer EDS, wavelength dispersion spectrometer WDS and electron backscattered diffraction EBSD). It enabled a comprehensive chemical composition and crystal orientation analyses.

The phase composition of coatings was analyzed by X-ray diffraction using the Philips PW 1830 diffractometer system. The tests were performed using filtered CoKα radiation within the 2θ angle from 30 to 75° in a step-scan mode with an angular step of 0.02° and counting time of 5 s.

Microhardness measurements were performed using the Vickers method. The microhardness was measured using the Microtech MX3 tester (Eybens, France) at a load of 0.4 N applied for 15 s. Indentations were made on perpendicular sections in three zones: in the coating (before and after LBP), in the heat affected zone (HAZ) and in the substrate material. Each sample was subjected to 10 measurements.

Measurements of the surface geometric structure (SGS) and roughness were carried out at the Laboratory of Computer Measurements of Geometric Quantities of the Kielce University of Technology. The tests were performed by means of the Talysurf CCI optical profiler using the patented Taylor Hobson’s coherence correlation algorithm, which enables measurements with an axial resolution of 10 pm. The test area and obtained horizontal resolution were 1.65 mm × 1.65 mm and 1.65 µm × 1.65 µm, respectively. 3-D images of the surface were analyzed with the TalyMap Platinium (version 6.2) software to determine the SGS of coatings.

The ESD WC-Co coatings were also subjected to a scratch test in order to determine their adhesion to the substrate. The measurements were performed with the CSM Instruments Revetest Xpress Scratch Tester. The scratch test parameters were as follows:

- Max. load-200 N;
- Table movement velocity-1 mm/min;
- Scratch length-5 mm;
- Rockwell diamond cone with a tip radius of 200 μm.

The scratch test principle is based on controlled scratching of a sample with a diamond indenter under changing loads. The normal force, friction force and penetration depth are continuously monitored. Moreover, an automated microscopic system analyzes the scratch lines along the whole length. It enables precise identification of the coating failure event. The lowest value of the normal force, which causes a loss of coherence between the coating and the substrate material is termed critical (F_cr_) and is regarded as the measure of adhesion.

The measurements of residual stresses in ESD WC-Co coatings were carried out before and after laser treatment using the Waisman–Phillips method based on the Sachs–Davidenkov theory. The Waisman–Phillips method was used for residual stress measurements. It is classified as a mechanical method consisting of chemical or electrochemical removal of successive layers of flat elements and measurements of changes in curvature caused by a release of residual stresses in the material.

Corrosion resistance tests were carried out by the computerized Atlas’99 electrochemical analysis system using the potentiodynamic method. The cathodic and anodic polarization curves were acquired by polarizing the tested specimens at 0.2 mV/s (within the area of ±200 mV from the corrosion potential) and 0.4 mV/s (within the area of higher potentials).

### 2.3. Experimental Design and Statistical Methods

The adhesion measurements were carried out in one-factor (F_cr_) two-level (as-coated and laser treated) experimental design. To reduce the inevitable influence of disturbing factors (noise originated from uncontrolled environmental factors, material, hardware and operator), 5 repetitions were made for each combination of settings. The mean values of the critical force measured on as-deposited and laser treated coatings were compared. The *t*-test for independent variables was applied [35]. In order to check whether the normality and equal variance assumptions were met, the Shapiro–Wilk and Levene’s procedures were applied [35].

The microhardness measurements were carried out in the two-factors mixed-level full factorial experimental design [36]: STAGE factor, assuming two settings (as-coated and laser treated) and the LOCATION factor, assuming three settings (coating, HAZ and substrate). To reduce the inevitable influence of disturbing factors (noise originated from environmental uncontrolled factors, material, hardware and operator), 10 repetitions were made for each combination of settings. In the case of microhardness, a two-way analysis of variance was performed [35] to assess the importance of particular factors and their interactions. Additionally, tests of mean equality [35] for particular locations were conducted to evaluate the impact of laser beam machining (LBM). Finally, principal component analysis [36] was performed to discover the real dimensionality of the dataset and possible data clusters.

## 3. Results and Discussions

### 3.1. Morphology

**Figure 4:**
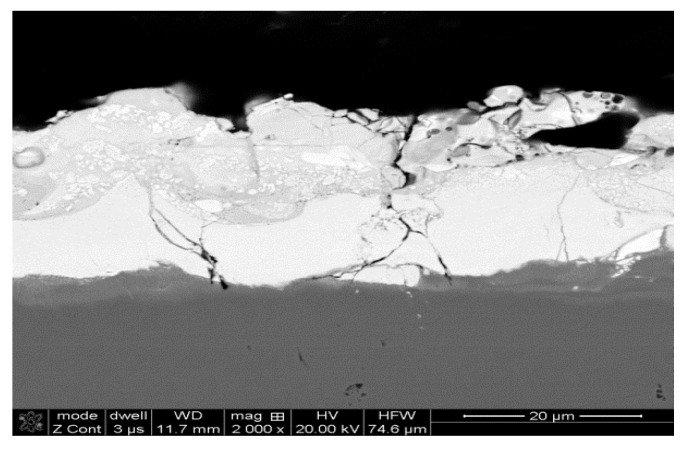
As-deposited microstructure of the WC-Co coating.

**Figure 5:**
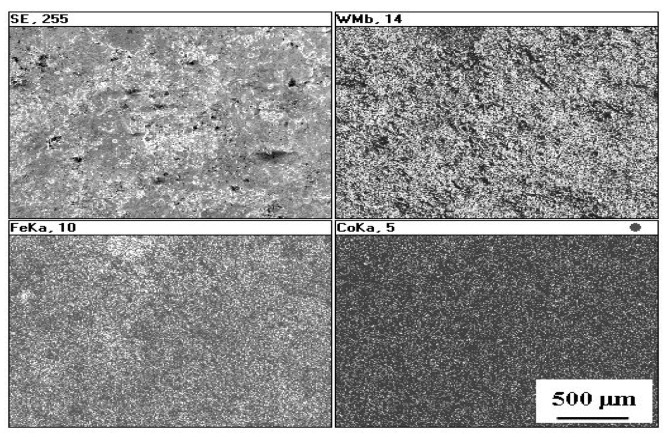
Distribution of elements on the WC-Co coating surface.

The microstructures of ESD WC-Co coatings in both as-deposited and laser-treated condition were observed by SEM. A typical microstructure of the WC-Co coating is illustrated in Figure 4. From the SEM analysis it is evident that the as-deposited coating was porous and cracked, and had a thickness of between 25 and 35 μm. The heat affected zone (HAZ) within the substrate ranged from 14 to 21 μm beneath the clearly seen coating–substrate interface. The ESD treatment homogenized the chemical composition of the coatings and refined their microstructure as shown in Figure 5.

**Figure 6:**
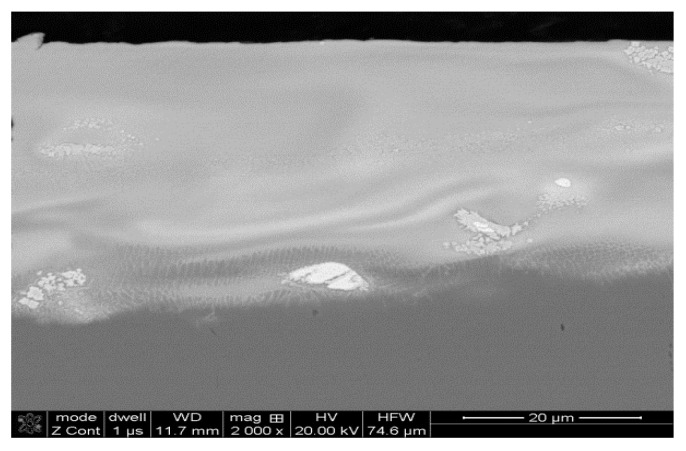
Microstructure of the WC-Co coating within the WC-Co coating after laser beam processing (LBP).

As seen in Figure 6, the laser-modified outer layer was free from cracks and porosity. The coating was 30–40 μm thick and perfectly adhered to the substrate, wherein the carbon-enriched HAZ extended from 25 down to 31 μm beneath the coating.

**Figure 7:**
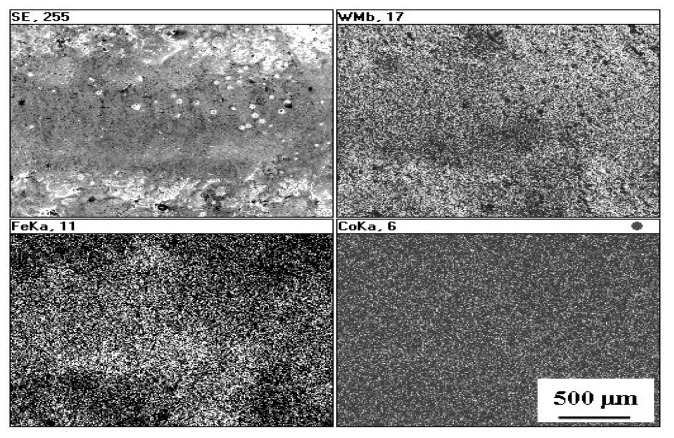
Distribution of elements on the WC-Co coating surface after LBP.

Distributions of elements in the WC-Co coatings before and after laser treatment are presented in Figure 5 and Figure 7. From Figure 7 it is evident that the laser treatment promoted formation of an iron-enriched layer in the center of the remelted coating.

**Figure 8:**
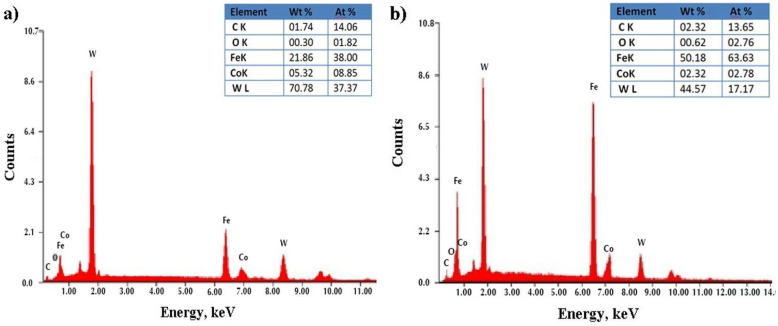
EDS analysis of the electro-spark deposited (ESD) WC-Co coatings: (**a**) as-deposited and (**b**) after LBP.

The EDS spectra of the WC-Co coating are illustrated in Figure 8. The presence of W, Co, C and Fe elements proved the alloy formation between the coating and the substrate. As can be seen, the coating surface was characterized as an irregular and rough view, due to the globular mass transfer mechanism during the ESD process from the electrode to the substrate. The EDX analysis of the WC-Co coating revealed that the laser treatment resulted in small changes in its composition (Figure 8b). The compositions of the coating before and after laser treatment were similar. The most intense peaks originated from W (Figure 8a,b).

### 3.2. X-ray Analysis

**Figure 9:**
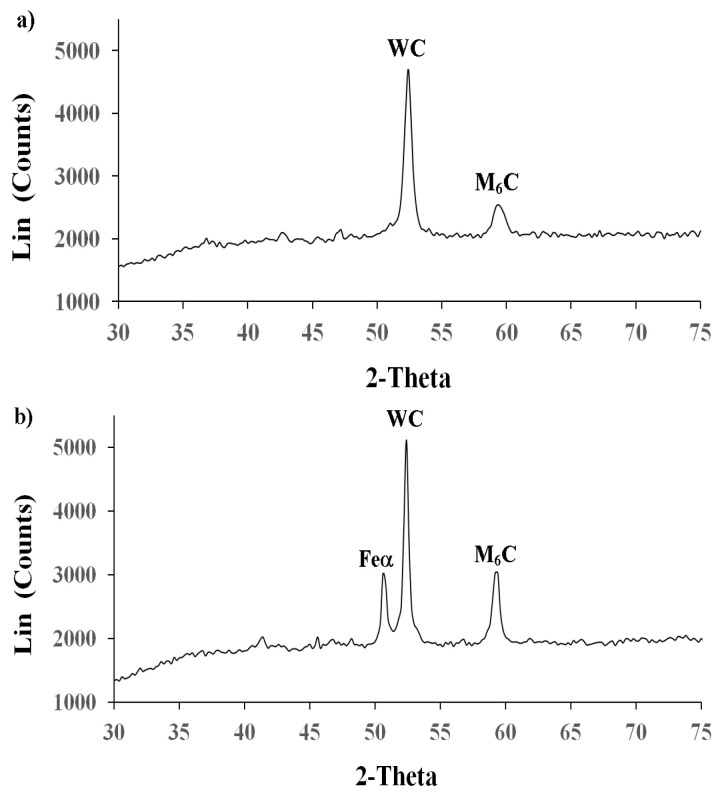
XRD patterns of the WC-Co coatings: (**a**) before LBP and (**b**) after LBP.

The phase composition analysis of the WC-Co coatings revealed that the surface layer of the coating consisted mainly of WC with small amount of M_6_C carbides, such as (Fe,Co,W)_6_C, both before and after LBP (Figure 9). The LBP rendered the WC-Co coating to melt together with part of the substrate, which resulted in the formation of ferrite and higher amounts of complex M_6_C carbides (Figure 9b). From Figure 9a,b it was evident that the most intense peaks originated from tungsten carbide.

### 3.3. Measurements of the Surface Geometric Structure

Parameters of the surface geometric structure (SGS) were among the main surface characteristics, which influenced various processes occurring within the subsurface layer. Refs [37,38] have been devoted to important issues related to measurement methods and evaluation of surface roughness and waviness. Measurements of SGS were used in practical applications, to present a spatial image that allows a correct understanding of the nature of the surface. The analysis of the geometric structure of a surface consists of three stages: measurement made using the selected method, presentation of the surface and parametric evaluation of the surface.

**Figure 10:**
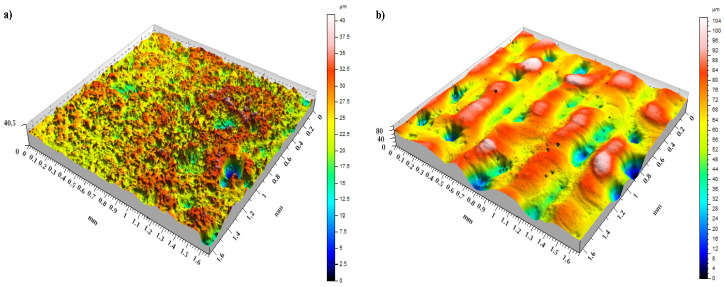
Topography of coatings: (**a**) before LBP and (**b**) after LBP.

**Figure 11:**
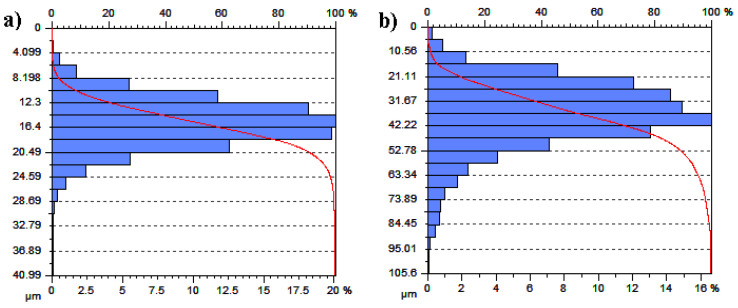
Distribution of ordinates and specimen bearing curves: (**a**) before LBP and (**b**) after LBP.

**Figure 12:**
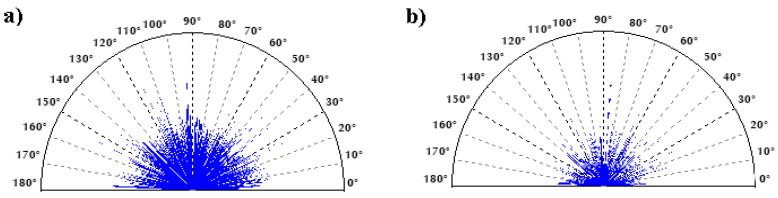
Isotropy of samples. (**a**) Before LBP and (**b**) after LBP.

Figure 10, Figure 11 and Figure 12 present data on surface characteristics, namely: surface topography (Figure 10), ordinate distributions with bearing curves (Figure 11) and isotropicity diagrams of samples (Figure 12)—all data are given before and after laser processing. Table 1 presents the most important parameters of the surface geometric structure of the tested samples.

The arithmetic mean of the coating surface height (*Sa*), i.e., the basic amplitude parameter for quantitative evaluation of the analyzed surface condition, more than tripled after LBP. A similar trend was observed for the mean square height of the surface (*Sq*). Additionally, the parameters: maximum peak height (*Sp*), maximum pit height (*Sv*) and maximum height (*Sz*) had more than 2 times higher values after laser treatment than before this treatment. The amplitude parameters, such as the surface asymmetric coefficient (*Ssk*) and surface slope coefficient (*Sku*), were used to characterize the surface shape. These parameters were sensitive to the occurrence of local hills or cavities on the surface, including defects. *Ssk* had a negative value for both surfaces, which indicates the presence of surfaces with plateau-shaped hills. The obtained values of slope of the surfaced close to each other and within the range *Sku* = 4.37–4.85 shows the ordinates distribution for both samples close to normal distribution.

Prior to LBP the coating surface had a random, isotropic structure (Iz = 86.47%), while after LBP it changed from isotropic to periodic, it fell into the transition zone between isotropic and anisotropic (Iz = 52.53%).

### 3.4. Roughness Measurements

The roughness of WC-Co coatings was measured in two perpendicular directions. The first measurement was made parallel to the electrode movement direction, while the second measurement was perpendicular to the scanning stitches. The average value of the Ra parameter for a given coating was calculated from these two measurements.

Measurements of WC-Co coatings subjected to LBP were made in the perpendicular and parallel direction to the path of laser beam, and then the mean value of roughness was calculated. In most research studies, the measurements of surface roughness were measured along the path of the laser beam. The obtained results did not reflect the actual surface microgeometry because the maximum height of irregularities occurs in the perpendicular direction.

**Figure 13:**
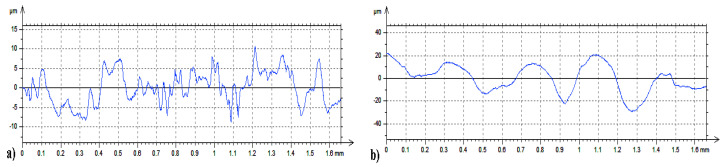
Examples of surface profiles of WC-Co coatings: (**a**) before LBM and (**b**) after LBM (perpendicular).

WC-Co coatings were characterized by the value of the parameter Ra = 2.64–3.16 μm, while after laser beam machining the arithmetic mean value of the profile ordinates ranged from 9.87 to 10.57 μm. C45 steel substrates to which coatings were applied were characterized by Ra = 0.38–0.42 μm. Selected profiles of the tested samples are presented in Figure 13.

From the obtained data it is evident that LBM increased the roughness of WC-Co coatings. The higher roughness of WC-Co coatings after laser treatment resulted from the movement of liquid metal caused by surface tension forces. The heterogeneous temperature distribution in the laser beam (mod TEM_00_) caused a heterogeneous surface profile after solidification, which, to a certain extent, reflected the energy distribution in the molten material.

In the case of pulse laser processing, it is assumed that the main factor determining the surface profile after solidification is the vapor pressure of the processed material, which causes “ejection” of the material from the central area and formation of characteristic outflows on both sides of the melted area. The influence of laser processing parameters on surface roughness was studied in detail in Refs [39,40,41].

### 3.5. Adhesion Tests

Friction force measurements and microscopic observations were used to determine the critical force (F_cr_) for WC-Co coatings deposited on C45 steel substrates. The obtained results are presented in Table 2.

**Figure 14:**
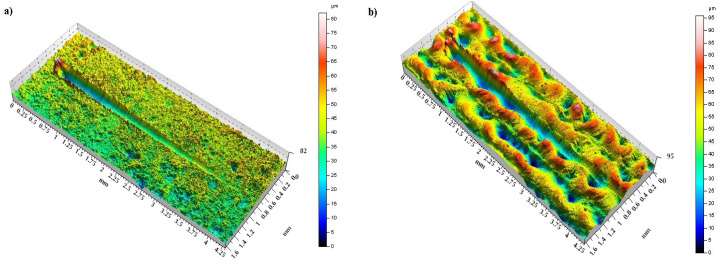
Three dimensional images of scratches made on the WC-Co coatings: (**a**) before LBP and (**b**) after LBP.

Examples of 3D images of scratches made using the Talysurf CCI optical profilometer are shown in Figure 14.

The results indicate that the LBP increased adhesion of WC-Co coatings to the C45 carbon steel substrate by around 30%. Moreover, the lower variation in critical force implies that laser processing eliminated defects located at the coating/substrate interface. 

**Figure 15:**
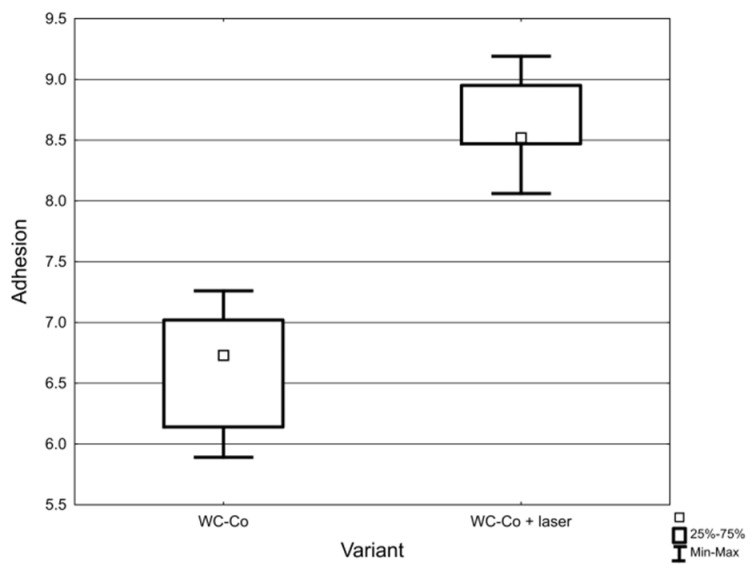
Raw data obtained from adhesion tests.

Both groups were subjected to significance tests to check the effect of LBM on F_cr_. Raw data is presented as a box plot in Figure 15.

The test of the mean equality of t was performed, obtaining the value *p* = 0.0003. This indicates that the difference between the mean values of F_cr_ was significant. It was also checked whether the test assumptions were met: (a) both groups passed the Shapiro–Wilk normality test with the *p*-level = 0.65 (WC-Co) and *p*-level = 0.85 (WC-Co + laser) and (b) Levene’s homogeneity of variance test gave the level *p* = 0.39. This means that the variances in both groups can be treated as equal.

### 3.6. Microhardness *Measurements*

**Figure 16:**
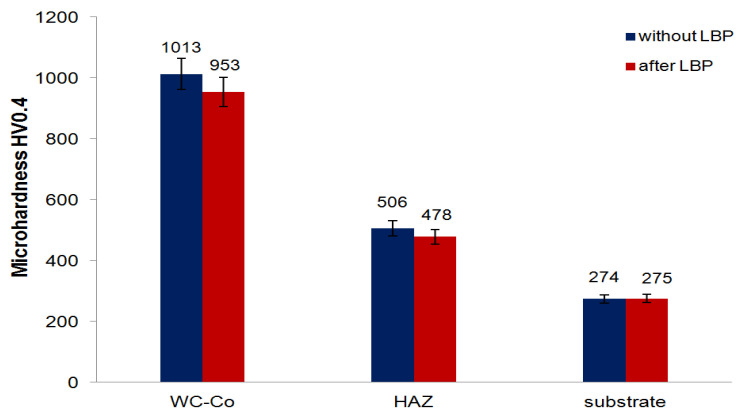
Microhardness measurement results.

It was found that LBP caused a slight decrease in microhardness of the tested coatings. The microhardness of the WC-Co coating prior to LBP was ranging from 968 to 1065 HV0.4 and slightly decreased to between 937 and 995 HV0.4 after LBP. The C45 steel substrate was not much affected by the laser treatment and its microhardness was ranging from between 461 and 528 HV0.4 in the HAZ to between 271 and 279 HV0.4 in the underlying substrate. Figure 16 compares the mean values of ten microhardness readings taken within the coating, the HAZ and in the substrate lying beneath the HAZ.

The slight decrease in microhardness of the tested coatings may potentially improve their ductility, which is crucial in applications where impact loading is involved, e.g., in drilling tools used in the mining industry or pressing dies used for construction ceramics manufacturing. This phenomenon can be the result of the dissolution of carbides. In the next stage of the experimental program, the parameters of the laser beam need to be set in such a way as to avoid the process of carbides’ dissolution in the shaped technological surface layer.

The obtained database was subjected to an analysis of variance (ANOVA) to check whether there were any statistically significant differences between the microhardness values obtained for the coating, HAZ and steel substrate. The results are presented in Table 3.

In order to assess the effect of LBP on microhardness in specific locations, tests of the equality of means in individual locations were carried out before and after laser treatment. The results are summarized in Table 4.

**Figure 17:**
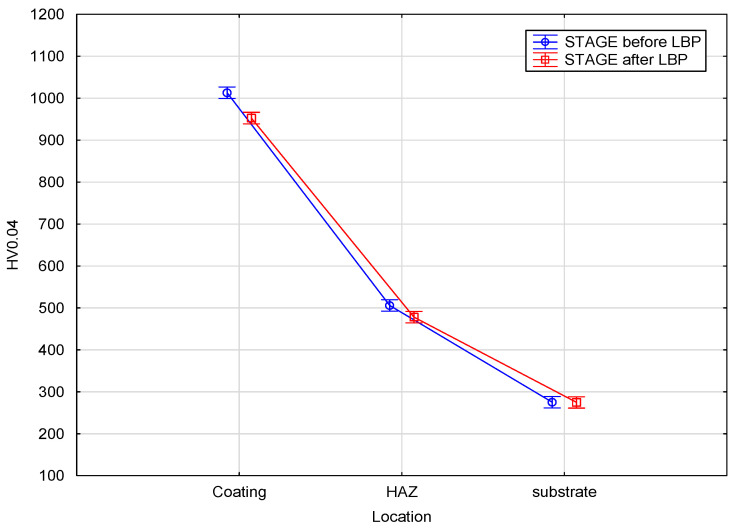
Comparison of the microhardness at particular locations before and after LBP.

The results of the analysis showed (Table 4 and Figure 17) that the laser processing had a significant effect on microhardness of the coating (*p*-value = 0.0009) and HAZ (*p*-value = 0.0005), and a negligible effect on the microhardness of the substrate (*p*-value = 0.75).

**Figure 18:**
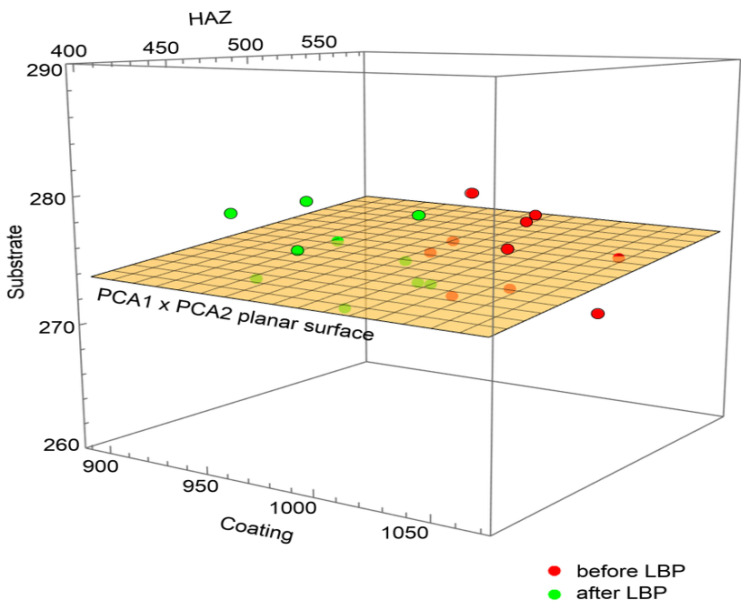
Comparison of microhardness at particular locations before and after LBP.

The 3D scatter plot allowed one to detect a clear clustering of the results (Figure 18), with the laser processing appearing to be the clearest differentiating factor.

**Figure 19:**
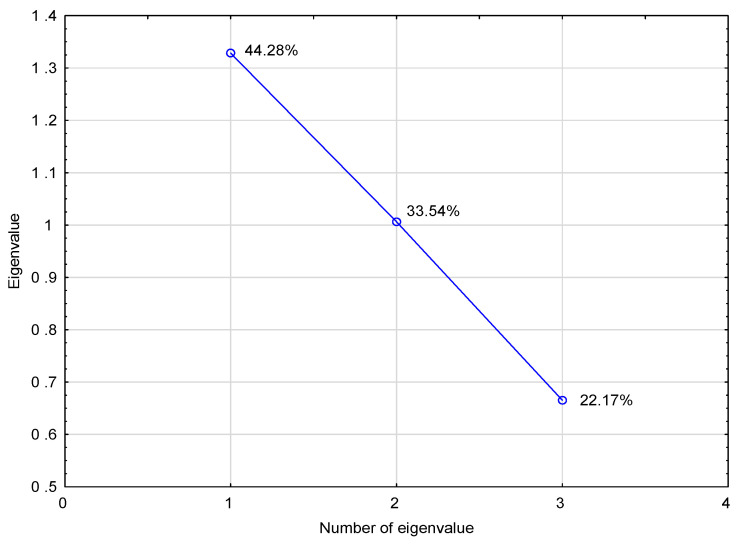
Scree plot for the microhardnesses dataset.

In order to unequivocally decide whether the visually observed grouping is not merely an artifact, principal components analysis was performed. The obtained scree plot (Figure 19) allowed one to assess that the formally three-dimensional (coating vs. HAZ vs. substrate) set of the microhardness data was predominantly a two-dimensional object, i.e., two dimensions explained 78% of variability. In practice, this means that the knowledge of two of these microhardnesses allows for the determination of the third component with high accuracy.

**Figure 20:**
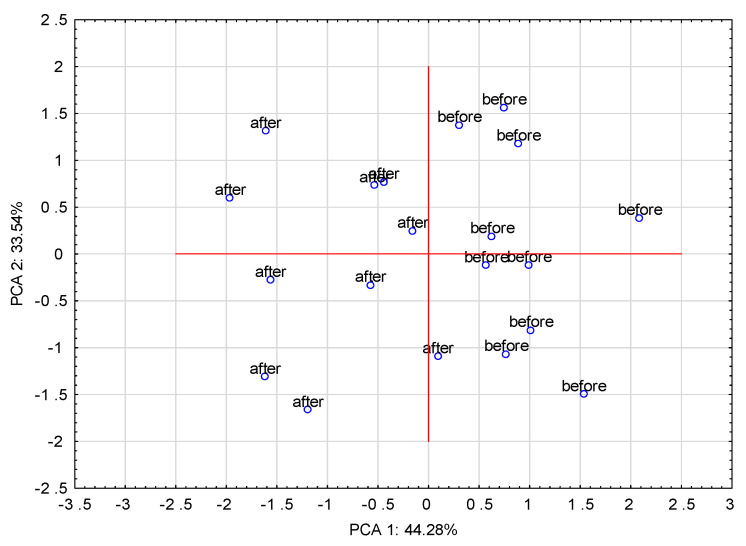
PCA1 vs. PCA2 plot revealing clustering of the microhardnesses dataset.

Clustering is clearly confirmed in the PCA1 vs. PCA2 (Figure 20), where the laser processing was expressed by the movement of a datapoint from the right area (before LBP) to the left area (after LBP) and these areas did not overlap. This revealed that the change in these properties was clear and significant.

### 3.7. Corrosion Resistance Tests

Potentiodynamic polarization curves were used to designate the corrosion potential (*E*_corr_) and corrosion current density (*j*_corr_). Extrapolation of Tafel lines is one of the most popular DC techniques for estimation of the corrosion rate. The extrapolation of anodic and/or cathodic Tafel lines for charge transfer controlled reaction gives the *E*_corr_ and *j*_corr_, at the corrosion potential. According to the Tafel’s law [42]:

*E* − *E*_0,c_ = *b*_c_ log (*j*_c_/*j*_0_) (1)

is the linear cathodic branch of polarization curve and:

*E* − *E*_0,a_ = *b*_a_ log (*j*_a_/*j*_0_) (2)

is the linear anodic branch of polarization curve. In Equations (1) and (2) *E*_0,c_, *E*_0,a_, *j*_0_, *b*_c_ and *b*_a_ are constant parameters characterizing polarization curves.

**Figure 21:**
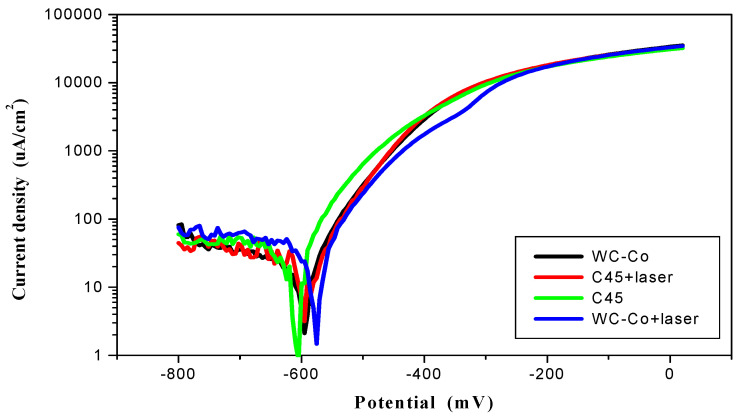
Polarization curves of the tested samples.

Specimens with a 10 mm diameter separated area were polarized to 500 mV. In order to establish the corrosion potential the polarization curves were acquired 24 h after exposure to the test solution (0.5 M NaCl). All tests were carried out at 21 ± 1 °C. The corrosion resistance results are shown in Figure 21.

After laser treatment of C45 carbon steel a martensitic structure was obtained, which has a higher resistance to corrosion as compared to the ferritic–pearlitic structure. LBP improved the corrosion resistance of ESD coatings by about 21% due to the sealing effect. As a result of laser processing a decrease in the corrosion current density, from 11.6 to 9.2 μA/cm^2^, and increase in the corrosion potential, from −595 to −585 mV, were observed. The characteristic electrochemical data are presented in Table 5.

The observed differences in potentials (between stationary and corrosive potentials) presumably resulted from the fact that during slow changes of the potential during testing (0.2 mV/s), it was possible to form hydroxides on the tested surface, which alkalized the environment in the vicinity of the sample and thus changed the potential of steel. It was assumed that a change in pH by a unit changed the corrosive potential by 50 mV. Moreover, the cathode processes were under diffusional oxygen reduction control and therefore the slope angles of the cathode curves of the tested samples had such high values.

### 3.8. Residual Stress Analysis

In the case of laser beam processing, thermal stresses and phase transition stresses depend mainly on the processing conditions and material properties. Tensile stresses arise after laser remelting of the surface material, while compressive stresses occur when laser treatment takes place without melting [43,44]. The energy density and the speed of the laser beam movement have the greatest influence on the type of resultant residual stress after laser processing [45].

**Figure 22:**
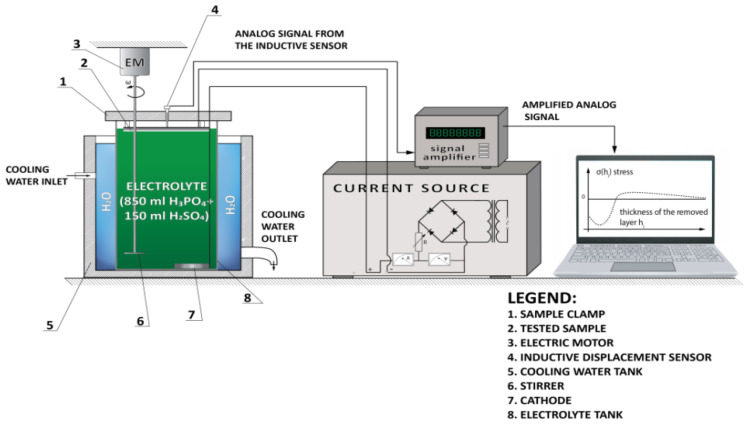
Experimental setup for measuring residual stresses using the Waisman–Phillips method.

A computerized setup for measuring residual stresses was used for the tests. Its schematic diagram with the data acquisition and processing systems is shown in Figure 22.

A concentrated acid solution including 850 mL of H_3_PO_4_ and 150 mL H_2_SO_4_ was used for electrochemical etching of the tested samples. The current density was 1 A/cm^2^.

**Figure 23:**
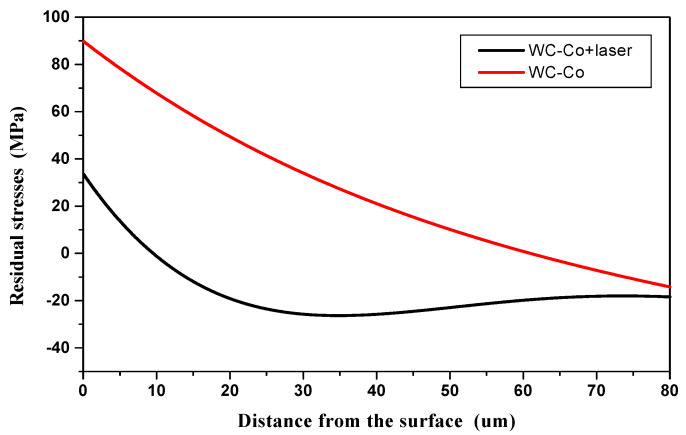
Distribution of residual stresses in the WC-Co coating in the as-coated and laser treated condition.

The tests were carried out for WC-Co coatings applied to C45 carbon steel rectangular samples 66 mm by 5 mm and 1.5 mm thick. The coated samples were tested both in the as-deposited and laser treated condition. In order to eliminate initial stresses in the steel substrates, which originate from plastic deformation by rolling, cutting, etc., they were subjected to annealing (stress relieving) for 12 h at 300 °C. The measurement results are presented in Table 6 and in Figure 23.

The measurements show that after deposition an unfavorable tensile stress of around 90 MPa arose at the surface of the coating. As presented in Figure 23, it had a tendency to decrease to zero with increasing distance from the surface to around 60 μm. Beyond this thickness the stress became compressive and reached −16 MPa at 80 μm distance from the surface.

The LBP reduced the magnitude of tensile stresses in the WC-Co coating by about 63% compared to the as-deposited state. Much lower tensile stress, of 33 MPa at the surface, converted to compressive stress at around 10 μm distance from the surface and reached −27 MPa at a distance of about 30 μm from the surface. It is assumed that the compressive stress may arise as a result of structural changes caused by phase transitions and should be related to the electrode and substrate materials used.

### 3.9. Field Performance of WC-Co Coatings

#### 3.9.1. Case Study 1

Regeneration of used gas bottle markers can be accomplished with a variety of techniques including application of WC-Co coatings. The markers are typically made of N11 tool steel. Their regeneration consisted of the application of WC-Co coating.

**Figure 24:**
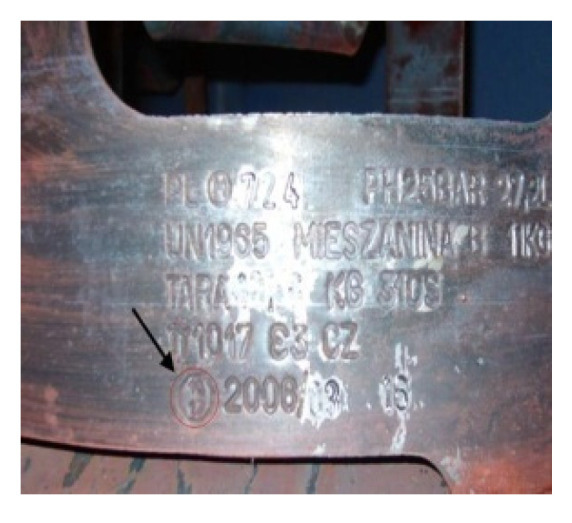
The flange of a gas cylinder with a permanent mark.

**Figure 25:**
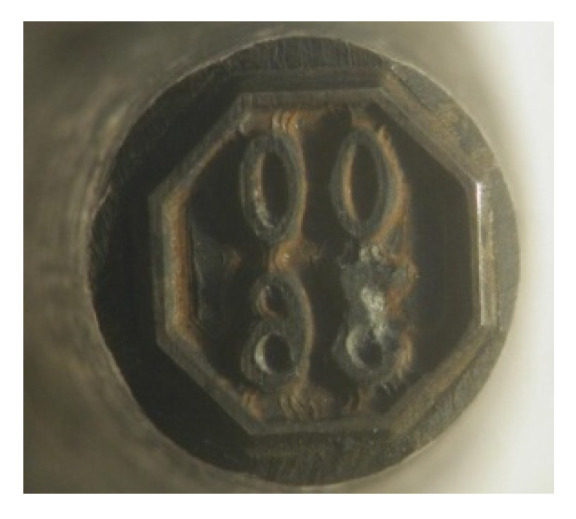
Worn marker intended for regeneration.

**Figure 26:**
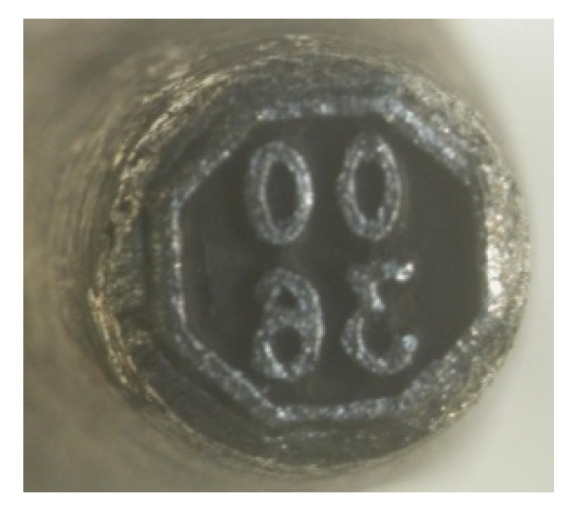
Marker with WC-Co ESD coating (without LBP).

As shown in Figure 24, permanent marks were applied to the cylinder flange during regeneration of used cylinders. Figure 25 and Figure 26 show a worn marker and a marker regenerated with the ESD WC-Co coating. As reported by Milmet S.A. Company (Sosnowiec, Poland), the markers coated/regenerated with the ESD WC-Co cermet coatings were tested under real working conditions and performed much better than their uncoated counterparts.

#### 3.9.2. Case Study 2

Comparative tests of uncoated and WC-Co EDS coated HS6-5-2 high speed steel (HSS) indexable inserts were conducted in an industrial environment at MESKO S.A Skarzysko-Kamienna. The machining involved lathe turning of 40H steel parts with Mecafluid 137 used to cool the tool and maintain swarf clearances.

**Figure 27:**
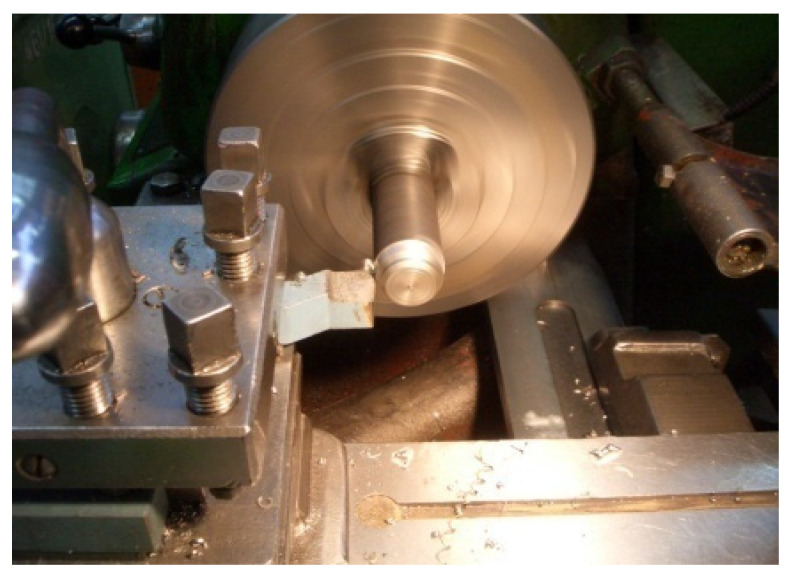
Lathe turning of 40H steel bars with an HS6-5-2 HSS indexable insert: cutting speed v_c_ = 57 m/min, workpiece rotation n = 450 rpm and feed *p* = 250 mm/min.

The criterion of edge blunting was assumed to be the shape of turning chips indicating the loss of cutting capacity and/or tool edge chipping. The turning operation and machining parameters are presented in Figure 27. The tests indicated that the average tool life increased from 189 to 438 min after replacing uncoated inserts that were WC-Co coated and laser processed ones.

#### 3.9.3. Case Study 3

**Figure 28:**
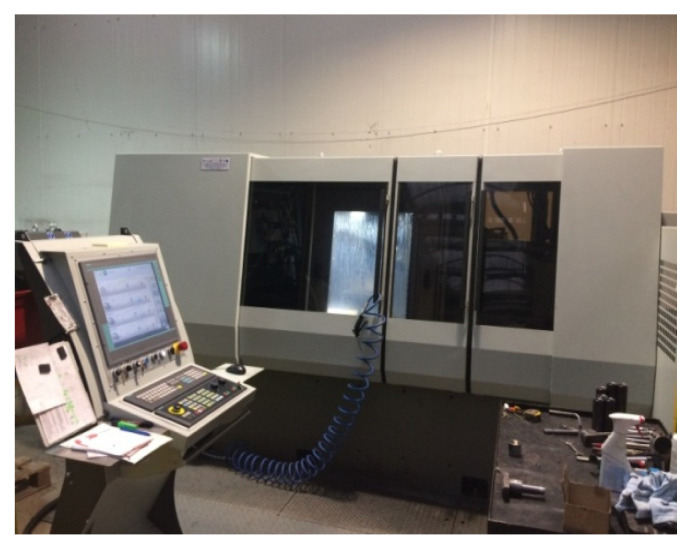
Computerised Numerical Control CNC lathe with a feeder.

**Figure 29:**
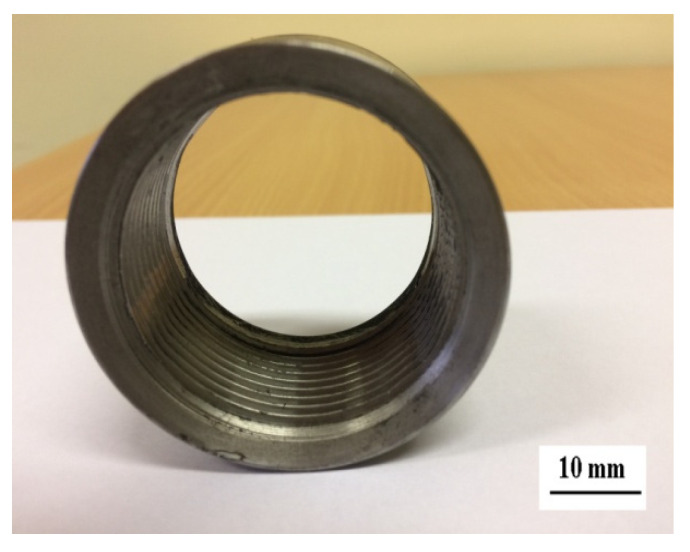
Joint with an inner thread.

Uncoated and WC-Co ESD coated and laser processed taps made of high-speed steel containing 8% cobalt (HSS-E) were subjected to industrial tests at Kiel-Inox, Kielce to compare their performance in machining of threads in central heating stove components made of S235JR steel. The tap durability tests were conducted on the Computerised Numerical Control CNC ADIGE-SYS EM80 lathe using the Emulkol PS coolant. The testing rig and processed component are shown in Figure 28 and Figure 29, respectively.

The industrial tests revealed markedly longer life of the coated tools. The average number of threaded components made with coated tools of WC-Co coating after LBP was 600 as compared to 110 pieces processed with uncoated taps.

## 4. Conclusions

The analysis and interpretation of the obtained test results enabled us to draw the following conclusions:

1. A concentrated laser beam could effectively modify the surface layer of ESD coatings.
2. The laser processed ESD WC-Co coatings show improved adhesion (by 24%) and resistance to corrosion (by 21%) and slightly decreased microhardness (of 6%).
3. Very low *p*-values corroborated statistical significance of the adhesion measurements and microhardness data obtained for the coating and HAZ.
4. The surface geometric structure parameters and roughness of the ESD WC-Co coatings more than tripled by the laser treatment.
5. The LBP eliminated deposition imperfections (pores, cracks, etc.) and slightly changed chemical and phase composition of the coating. The laser beam caused the WC-Co coating to melt and dissolve iron from the substrate, thus promoting formation of ferrite and complex M_6_C carbides within the coating.
6. The LBP markedly decreased the surface tensile stresses generated in the WC-Co coatings during ESD deposition and converted them into compressive ones in the zone lying deeper than 10 μm from the surface.
7. Electro-spark WC-Co coatings could be successfully used in the regeneration of tool components, such as gas cylinder permanent marks, and upgrading cutting/turning tools to a higher performance.
8. Further experimental studies ought to focus on testing the resistance to abrasive and erosive wear of ESD coatings before and after LBP.

## Figures and Tables

**Table 1:** Parameters of the surface geometric structure (SGS) of the WC-Co coating before and after laser beam processing.

Parameters	Layer	
	WC-Co	WC-Co + LBP
*Sp* (μm)	15.65	37.02
*Sv* (μm)	25.34	68.54
*Sz* (μm)	40.94	105.61
*Sa* (μm)	3.12	10.71
*Sq* (μm)	4.02	13.98
*Ssk*	−0.50	−0.80
*Sku*	4.85	4.37

**Table 2:** Scratch test results.

Layer	F_cr_ (N)					Mean (N)	Standard Deviation (N)
	Measurement Number						
	1	2	3	4	5		
WC-Co	6.14	5.89	7.26	6.73	7.02	6.61	0.58
WC-Co + laser	8.47	8.06	9.19	8.52	8.95	8.64	0.44

**Table 3:** Two-way ANOVA of the microhardness dataset.

Source	SS	df	MS	F	*p*
const	20,410,834	1	20,410,834	44,136	0.000000
STAGE	13,113	1	13,113	28.36	0.000002
LOCATION	5,261,729	2	2,630,864	5689	0.000000
interaction	9053	2	4527	9.79	0.000236
Error	24,972	54	462		

**Table 4:** Tests of microhardness equality in particular locations.

Location	Mean1	Mean2	t	df	*p*
Coating	1013.1	952.6	3.97	18	0.0009
HAZ	505.9	478.1	4.23	18	0.0005
Substrate	275.1	274.7	0.32	18	0.7513

**Table 5:** The values of corrosion parameters of the tested samples.

Material	Corrosion Potential (mV)	Corrosion Current Density (µA/cm^2^)	−b_c_ (mV/dec)	b_a_ (mV/dec)
C45	−630	15.9	605	90
C45 + laser	−605	14.4	495	90
WC-Co	−595	11.6	440	75
WC-Co + laser	−585	9.2	410	100

**Table 6:** Results of measurements of residual stresses.

Coating	Residual Stresses	
	Max. Tensile Stresses-σt (MPa)	Max. Compressive Stresses-σc (MPa)
WC-Co	90	−16
WC-Co + laser	33	−27

## Data Availability

The data presented in this study are available on request from the corresponding author.
